# Area under the curve analysis of blood pressure reveals increased spontaneous locomotor activity in SPAK knock‐in mice: relevance for hypotension induced by SPAK inhibition?

**DOI:** 10.14814/phy2.13997

**Published:** 2019-02-03

**Authors:** Kieran Burgess, Sofija Jovanović, Rajni Sudhir, Aleksandar Jovanović

**Affiliations:** ^1^ Division of Molecular and Clinical Medicine Medical School University of Dundee Dundee UK; ^2^ University of Nicosia Medical School Nicosia Cyprus; ^3^ Center for Neuroscience and Integrative Brain Research (CENIBRE) University of Nicosia Medical School Nicosia Cyprus

**Keywords:** Blood pressure, hypertension, locomotor activity, SPAK

## Abstract

SPAK (Ste20/SPS1‐related proline/alanine‐rich kinase) has been recently identified as a protein kinase which targets the electroneutral cation‐coupled chloride cotransporters and it stands out as a target for inhibition in novel anti‐hypertensive agents. From this prospective, any information about physiological consequences of SPAK inhibition would be useful to better understand the efficacy and potential adverse effects of the SPAK‐based anti‐hypertensive therapy. Radiotelemetry was employed to continuously measure the parameters of blood pressure (mean arterial blood pressure, systolic blood pressure, and diastolic blood pressure), heart rate, and physical activity in SPAK knock‐in (KI) mice and littermate controls for 24 h. For each parameter, the area under the curve (AUC) was calculated and compared between the SPAK KI mice and wild type mice. There was no statistically significant difference in the AUC of blood pressure parameters between SPAK KI and littermate mice. When mice were physically inactive, the AUCs for blood pressures were significantly lower in SPAK KI than in littermates. When physically active, blood pressures were significantly higher in SPAK KI than in littermates. The heart rate followed a similar pattern. The AUC of physical activity was significantly increased in SPAK KI mice when compared to littermates and the SPAK KI mice spent significantly less time in an inactive state and significantly more time in active states. Comparison between SPAK KI mice and unrelated wild type mice yielded similar results to the comparison between SPAK KI mice and littermates. We conclude that SPAK inhibition increases spontaneous locomotor activity, which has a significant effect on blood pressure.

## Introduction

SPAK (Ste20/SPS1‐related proline/alanine‐rich kinase) is a protein kinase which targets the electroneutral cation‐coupled chloride cotransporters (SLC12), which are established targets for anti‐hypertensive agents such as loop and thiazide diuretics. A genome‐wide association study (Wang et al. 2009) showed that intronic single nucleotide polymorphisms within the SPAK gene, which could be linked to 20% of the population, resulted in significantly increased blood pressure. SPAK is in the germinal center kinase‐VI (GCK‐VI) subfamily, along with a very similar kinase, oxidative stress response kinase (OSR1). Both SPAK protein and mRNA transcripts are found in many sites around the body, including but not limited to the skeletal muscle, testis, pancreas, lung, kidney, heart, and the brain (Murthy et al. 2017; Shekarabi et al. 2017).

SPAK and OSR1 phosphorylate sodium‐chloride symporter (NCC) and Na–K–Cl cotransporter 2 (NKCC2) on N‐terminal cytoplasmic serines and threonines (Thr46, Thr55, and Thr60 are phosphorylation sites in human NCC, and in NKCC2 the sites are Thr95, Thr100, and Thr105) in the kidney, which activates the cotransporters. These have key roles in renal salt reabsorption and extracellular fluid volume, which in turn affects blood pressure. To influence the cotransporters, SPAK and OSR1 must be activated by With No lysine = K (WNK) kinases (Richardson et al. 2011). SPAK knock‐in (KI) mouse model expresses a form of SPAK that cannot be activated by WNKs, resulting in a significant reduction in blood pressure (Rafiqi et al. 2010). Thus, WNK kinases modulate the activity of NCC in the DCT through a SPAK‐dependent mechanism. SPAK is therefore essential for the phosphorylation and activity of NCC, and this information, coupled with established links of several single nucleotide polymorphisms in the SPAK gene to hypertension, shows that SPAK has a key role in blood pressure regulation (Alessi et al. 2014).

The SPAK‐WNK protein complex stands out as a target for inhibition in novel anti‐hypertensive agents. Recently, many compounds have been tested as SPAK/OSR1/WNK kinase inhibitors and suggested as potential therapy for arterial hypertension (Cohen and Alessi 2013; Yamada et al. 2016; AlAmri et al. 2017).

From this prospective, any information about physiological consequences of SPAK inhibition would be useful to better understand the efficacy and potential adverse effects of the SPAK‐based anti‐hypertensive therapy. Here, we have employed telemetry on SPAK knock‐in (KI) mice to analyze blood pressure over a 24‐h period. We found out that lower SPAK activity is actually associated with increased spontaneous locomotor activity that counteracts the hypotensive effect of SPAK inhibition.

## Methods

### Mice

The SPAK KI and wild type littermate control mice described in Rafiqi et al. (2010) were used in the study. These mice were maintained on an inbred C57BL/6J background. In a separate series of experiments, wild type mice unrelated to SPAK mice of mixed CBAxBalb/c and C57BL/6 J background were used (phenotypes described in Du et al. 2006; Sudhir et al. 2011). All mice were on the same standard diet ad libitum. Experiments were approved by the Home Office and carried out as stipulated in Project Licenses 60/3152 and 60/3925.

### Surgical implantation of blood pressure radiotransmitters in SPAK KI and littermates

Before the implantation, the mice were anesthetized with inhaled isoflurane using a precision vaporizer within an induction chamber. The left carotid artery was initially isolated and the tip of the telemeter catheter (transmitter model TA11PA‐C10, Data Sciences International, St. Paul, MN) was inserted into the carotid artery and then advanced into the aortic arch. The body of the telemeter was positioned in a subcutaneous pocket on the right flank. All surgeries have been performed at the same time of the day (completed at 4 pm). Following the procedure, each animal was returned to its home cage, where they stayed in unrestricted solitary conditions. They were all provided with water and ad libitum standard mouse food for the duration of assessment. The telemeter signal was processed using RPC‐1 receiver, a 20‐channel data exchange matrix, APR‐1 ambient pressure monitor, and a Data Quest ART Gold 3.0 acquisition system (Data Sciences International, St. Paul, MN). The system was set to record the mean arterial, systolic, diastolic, and pulse pressure measured in millimeters of mercury (mmHg), heart rate (HR) measured in beats per minute (bpm), and locomotor activity (spontaneous physical activity/PA), which was measured in arbitrary units (AU) in increments of 6 and averaged over a 10‐sec interval. The average values of these parameters within this interval were calculated. Physical activity was measured by the horizontal displacement of the mouse in relation to two antennas in the receiver situated under the respective cage. In each of the mice, the data recordings initiated immediately postoperatively and continued continuously for at least 10 days. The data included in the analysis were from the monitoring of 3 knock‐in and 3 wild type mice. The recording room was maintained at 21–22°C with a 12:12 h light dark cycle (6 pm–6 am night and 6 am–6 pm day with 5.30–6 am dawn).

### Surgical implantation of ECG radiotransmitters in SPAK KI unrelated wild type

Mice were anesthetized using a constant flow of oxygen and isoflurane into a whole body chamber (Harvard Apparatus). The peritoneal cavity was exposed and the transmitter was placed flat side down in the abdominal cavity (ETA‐F20, Data Sciences International, St. Paul, MN). A needle was used to feed the leads of the transmitter from the abdominal cavity into the peritoneal cavity. The transmitter was then anchored in place by incorporating the grooves present on the transmitter body into the stitch line that sealed the abdominal cavity. The electrodes were then positioned on the body, with the positive electrode being positioned on the left xiphoid process and the negative electrode being positioned on the right shoulder of the mouse. The incision was closed with surgical staples. The system was set to record the locomotor activity (spontaneous physical activity/PA) and the ECG over a 10‐sec interval and calculate the average values of these parameters within this period. Physical activity was measured by the horizontal displacement of the mouse in relation to two antennas in the receiver situated under the respective cage. In each of the mice, the data recordings initiated immediately postoperatively at 4 pm following reintroduction to cage, and continued for at least 10 days. The data included in the analysis were from the monitoring of 4 of these mice. The recording room was maintained at 21–22°C with a 12:12 h light dark cycle (6 pm–6 am night and 6 am–6 pm day with 5.30–6 am dawn).

### Data analysis and statistics

The readings from day 6 to 7 were selected for analysis to allow for the mice to habituate to the environment. As the data were averaged every 10 sec, this comprised 8640 data points for every mouse for physical activity, heart rate, mean arterial pressure, systolic pressure, and diastolic pressure. For some parameters (mean arterial pressure, heart rate, and locomotor activity), mean values and standard error of the mean (SEM) of 8640 data points per mouse have been calculated. To assess the effect of diurnal rhythm of these parameters, we have analyzed data from midnight to 4 am (night) and from noon to 4 pm (day). For each parameter, the area under the curve (AUC) was calculated using SigmaPlot 12.5 Program (Jandel Scientific, Chicago, IL). The data from day 6 to 7 for physical activity were isolated then first used to construct line graphs for the mean physical activity of the knock‐in and wild type mice using SigmaPlot 12.5 software (Jandel Scientific, Chicago, IL). The data of the physical activity were also used to calculate the average time spent in both the active and inactive states of each group of mice. Any level of movement recorded from the telemetry device was defined as activity, so all the data points measuring 0 were deemed to be the inactive state, as no level of movement was recorded via the receiver. Any readings other than 0 were considered active. Data are presented as mean ± standard error of the mean (SEM), and *n* represents the number of analyzed mice. When calculating averages, data were not aggregated, but a single data point was calculated for each mouse (average of averages). Mean values of parameters were compared with the Student's *t*‐test. The statistical analysis was carried out using SigmaPlot 12.5 Program. *P* < 0.05 was considered statistically significant.

## Results

### SPAK knock‐in mice have trend toward lower average blood pressure with preserved diurnal rhythm

**Figure 1:**
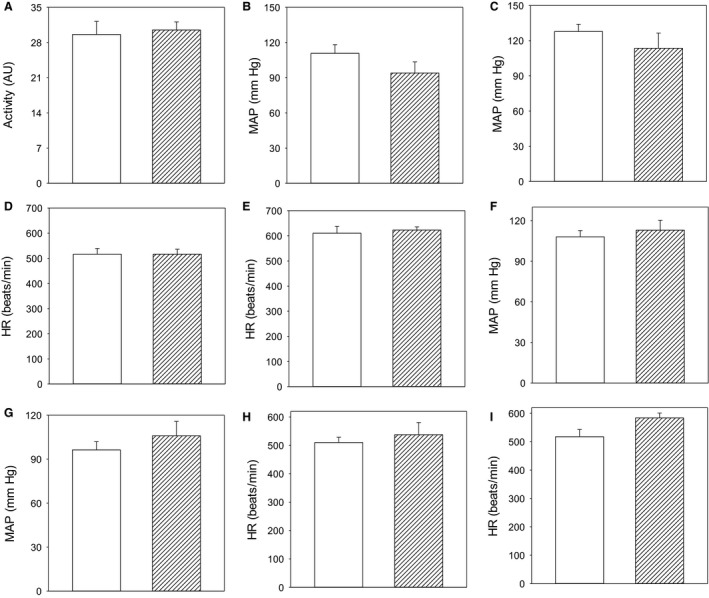
A trend in SPAK knock‐in mice toward lower mean arterial blood pressure while the locomotor activity, heart rate, and diurnal rhythm are not affected at all. (A–E) Bar graphs depicting the locomotor activity (A), mean arterial pressure (B and C), and heart rate (D and E) when mice were physically inactive (B and D) and active (C and E). Open and line bars correspond to wild type and SPAK KI, respectively. Each bar represents the mean ± SEM (3 animals were analyzed per group). F–I. Bar graphs showing the mean arterial pressure (F and G) and heart rate (H and I) during day (open bars) and night (line bars) in wild type (F and H) and SPAK KI (G and I). Each bar represents the mean ± SEM (3 animals were analyzed per group).

It has been reported that SPAK KI mice have lower blood pressure when compared to WT (wild type) when measured by both telemetry and tail‐cuff method (Rafiqi et al. 2010). Here, we have compared the average mean values of mean arterial pressure, heart rate, and locomotor activity between mice. In SPAK KI mice, there was a trend toward lower blood pressure although statistical significance was not reached (*P* = 0.118, *n* = 3; Fig. 1). On the other hand, no trend or statistically significant difference was observed in the heart rate (*P* = 0.316, *n* = 3; Fig. 1) or locomotor activity (*P* = 0.391, *n* = 3; Fig. 1). Diurnal rhythms of mean arterial pressure and heart rate have been observed in both WT and SPAK mice (Fig. 1).

### AUC analysis does not reveal lower blood pressures in SPAK KI mice

**Figure 2:**
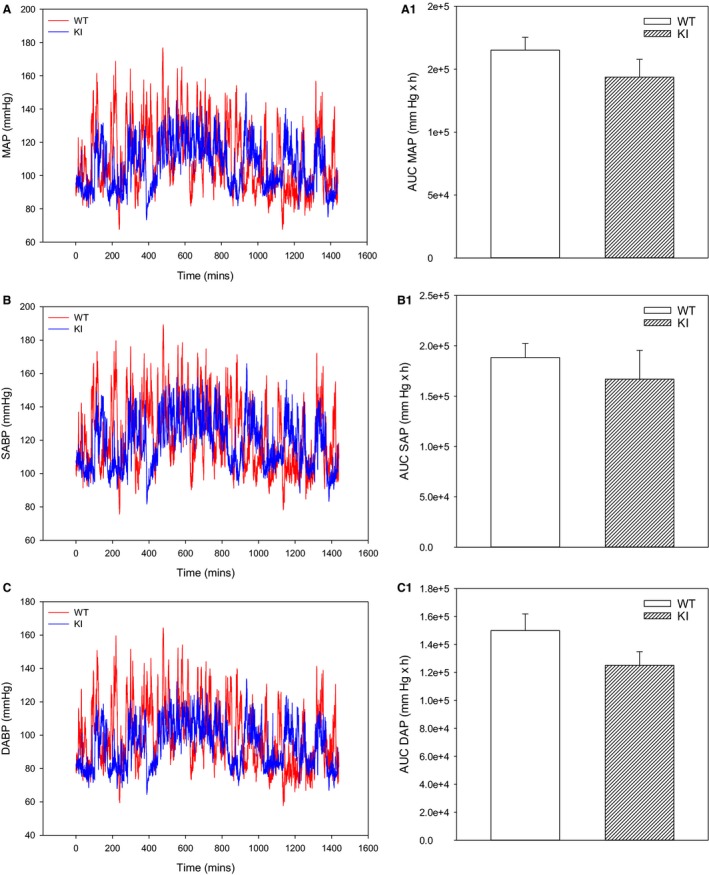
SPAK knock‐in mice do not have lower blood pressures over 24 h. Original time courses of mean arterial pressure (A), systolic arterial pressure (B), and diastolic arterial pressure (C), and corresponding area under the curve (AUC) bar graphs in SPAK KI mice and littermate wild type mice (WT). Each bar represents the mean ± SEM (3 animals were analyzed per group).

Telemetry measurements provided the time course of mean arterial blood pressure, systolic blood pressure, and diastolic blood pressure in freely moving conscious SPAK KI mice and littermate controls over 24 h (Fig. 2A–C). There was no statistically significant difference in the AUC of these parameters between SPAK KI and littermate mice (Fig. 2A1, B1, and C1).

### AUC analysis reveals lower blood pressures in SPAK KI mice when inactive

**Figure 3:**
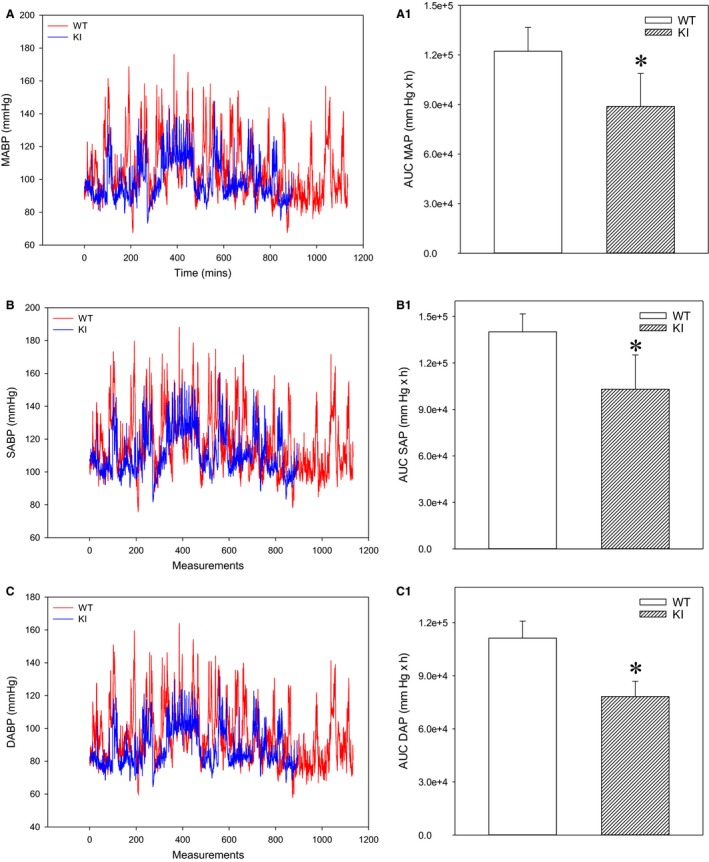
SPAK knock‐in mice have lower blood pressures over 24 h when inactive. Original time courses of mean arterial pressure (A), systolic arterial pressure (B), and diastolic arterial pressure (C), and corresponding area under the curve (AUC) bar graphs in SPAK KI mice and littermate wild type mice (WT). Each bar represents the mean ± SEM (3 animals were analyzed per group). *P < 0.05.

When values for blood pressures in inactive state alone were extracted and selected (Fig. 3A–C), AUCs for mean arterial blood pressure, systolic arterial blood pressure, and diastolic arterial blood pressure were significantly lower in SPAK KI mice when compared to littermates (Fig.  3A1, B1, and C1).

### AUC analysis reveals higher blood pressures in SPAK KI mice when active

**Figure 4:**
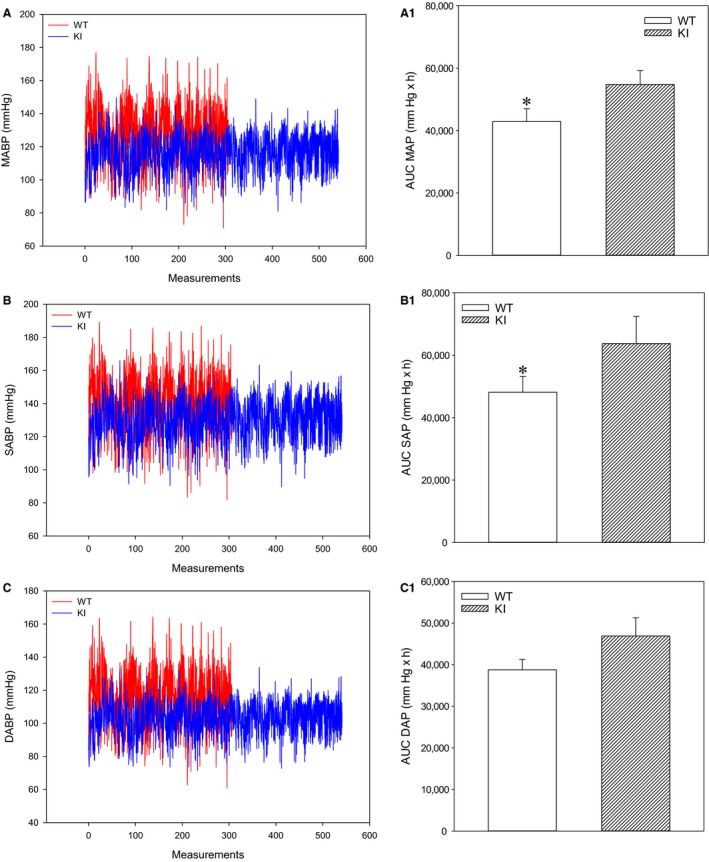
SPAK knock‐in mice have higher blood pressures over 24 h when active. Original time courses of mean arterial pressure (A), systolic arterial pressure (B), and diastolic arterial pressure (C), and corresponding area under the curve (AUC) bar graphs in SPAK KI mice and littermate wild type mice (WT). Each bar represent mean ± SEM (3 animals were analyzed per group). *P < 0.05.

When values for blood pressures in active states were extracted and selected (Fig. 4A–C), AUCs for mean arterial blood pressure and systolic arterial blood pressure were significantly different (Fig. 4A1, B1, and C1). Mean arterial blood pressure and systolic blood pressure were significantly higher in SPAK KI mice when compared to littermates.

### AUC analysis increased heart rate in SPAK KI mice when active

**Figure 5:**
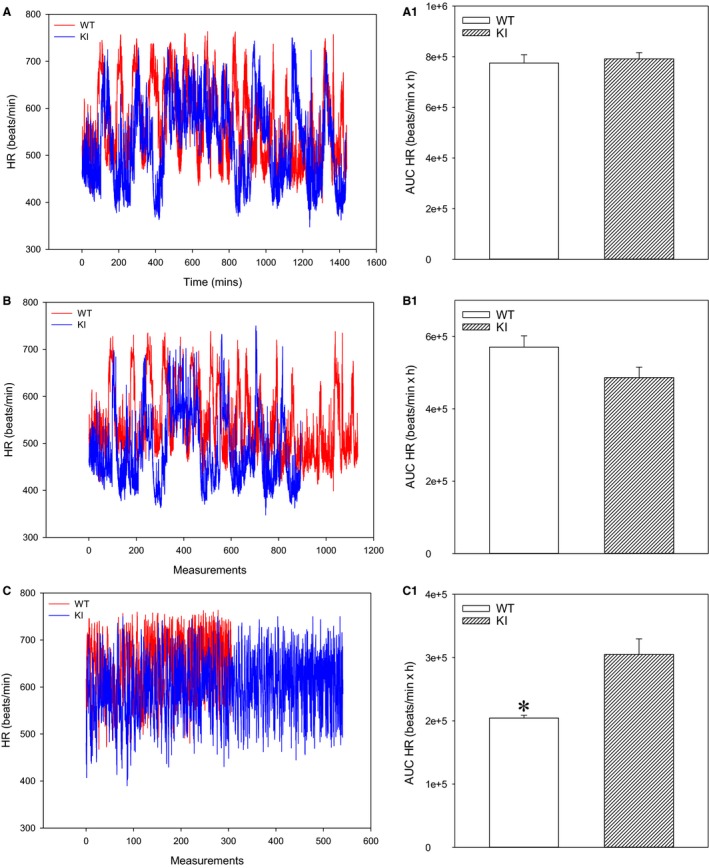
Heart rate is increased in SPAK knock‐in mice over 24 h when active. Original time courses of heart rate over 24 h irrespective of activity (A), when inactive (B) and when active (C), and corresponding area under the curve (AUC) bar graphs in SPAK KI mice and littermate wild type mice (WT). Each bar represents the mean ± SEM (3 animals were analyzed per group). *P < 0.05.

Heart rate AUC was not significantly different between SPAK KI and littermates over 24 h and when mice were inactive (Fig. 5BA‐1). However, heart rate AUC was significantly increased in SPAK KI mice when they were active (Fig. 5C, C1).

### SPAK KI mice spend more time in physically active state than WT

**Figure 6:**
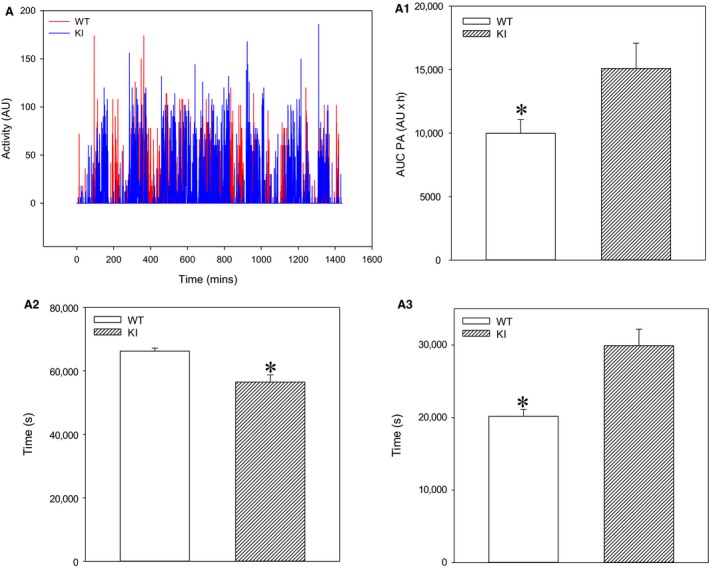
SPAK knock‐in mice have increased spontaneous locomotor activity when compared to littermate wild type mice. Original time courses of physical activity over 24 h (A) and corresponding area under the curve (AUC; A1), time spent in inactive state (A2), and time spent in active state (A3) bar graphs in SPAK KI mice and littermate wild type mice (WT). Each bar represents the mean ± SEM (3 animals were analyzed per group). *P < 0.05.

**Figure 7:**
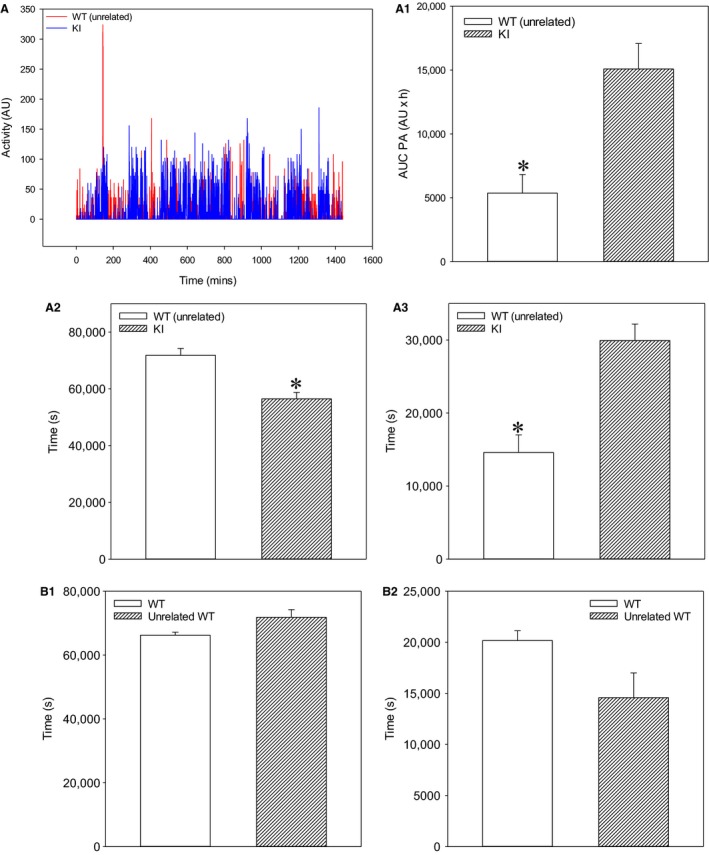
SPAK knock‐in mice have increased spontaneous locomotor activity when compared to unrelated wild type mice (A–A3). Original time courses of physical activity over 24 h (A) and corresponding area under the curve (AUC; A1), time spent in inactive state (A2), and time spent in active state (A3) bar graphs in SPAK KI mice and unrelated wild type mice (WT). Each bar represents the mean ± SEM (3 SPAK KI mice and 4 unrelated WT were analyzed). *P < 0.05. SPAK KI littermate wild type mice and SPAK KI‐unrelated wild type mice do not have different levels of physical activity (B1–B2). Bar graphs depict time spent in inactive state (B1) and time spent in active state (B2) in SPAK KI littermate wild type mice (WT) and SPAK KI‐unrelated wild type mice (unrelated WT). Each bar represents the mean ± SEM (3 SPAK KI mice and 4 unrelated WT were analyzed).

As physical activity is an important regulator of blood pressure and heart rate, we have examined whether spontaneous locomotor activity between SPAK KI and littermates differs. We have found that the AUC of physical activity was significantly increased in SPAK KI mice when compared to littermates (Fig. 6A, A1). The SPAK KI mice spent significantly less time in inactive state (Fig. 6A2) and significantly more time in active states (Fig. 6A3). To further examine a possibility that SPAK inhibition increases spontaneous locomotor activity, we have compared the physical activity of SPAK KI mice with those in wild type mice unrelated to SPAK KI mice. Comparison between SPAK KI mice and unrelated wild type mice yielded similar results as the comparison between SPAK KI mice and littermates (Fig. 7). No significant difference in spontaneous locomotor activity was observed between wild types of different background (Fig. 7).

## Discussion

The importance of SPAK and its role in regulating blood pressure via WNK kinases has previously been published in many papers (Richardson et al. 2011; Shekarabi et al. 2017; Murthy et al. 2017). It has been previously found that knock‐in mice that express a SPAK form that is unable to be activated by WNK kinases are significantly salt‐wasting and hypotensive (Rafiqi et al. 2010). The inhibition of the SPAK‐dependent signaling pathway has been proposed as an exciting prospect in therapy of hypertension (Cohen and Alessi 2013; Alessi et al. 2014; Yamada et al. 2016; AlAmri et al. 2017).

In the present study, when we analyzed data in a traditional way, we have confirmed previous findings that SPAK KI mice are hypotensive without any differences being observed in the physical activity and heart rate (Rafiqi et al. 2010).

Telemetry provides the measurement of blood pressures under “real‐life” conditions where this parameter is monitored in awake and freely moving laboratory animals. This allows insight into blood pressure under physiological conditions as it has never been seen before (Huetteman and Bogie 2009). Such long‐lasting continuous telemetric recordings produce data that can be used to calculate the AUC, which has been suggested to be the most appropriate parameter to investigate the 24‐h ambulatory blood pressure (O'Brien et al. 2013). The AUC analysis showed that there was no difference in mean, systolic, and diastolic arterial blood pressures between SPAK KI and littermate mice. However, the present study is the first one to implement AUC analysis of uninterrupted 24 h‐long telemetry recordings and no previous study has analyzed such data. Thus, as SPAK is a well‐established regulator of blood pressure, it was unclear what factors could counteract hypotension induced by SPAK inhibition. It has previously been reported that the 24‐h pattern of blood pressure is hemodynamically mediated primarily by the 24‐h pattern of cardiac output, and this pattern of cardiac output is almost entirely determined by the 24‐h pattern of heart rate (Kurtz et al. 2014). Data we obtained with the heart rate were in accord with those obtained for blood pressure. In the inactive state, there was no significant difference in the heart rate between phenotypes, but SPAK KI mice had significantly increased heart rate when physically active, which was associated with an increase in systolic, diastolic, and mean arterial pressures. The results obtained on the heart rate and blood pressures corresponded to each other and were in line with the well‐established notion that blood pressure follows the cardiac output pattern (Kurtz et al. 2014).

Physical activity is known to regulate blood pressure and heart rate (Perez‐Quilis et al. 2017). Here, we found that blood pressures in SPAK KI were lower when mice were inactive and higher when mice were physically active than those in WT. The level of physical activity of mice did not differ, but SPAK KI mice spent longer in the physically active state when compared to WT. This suggests that physical activity‐dependent changes in blood pressure were due to differences in times that SPAK KI and WT littermates spent in inactive/active states. This would imply that SPAK inhibition regulates the locomotor activity. However, when considering that we used small number of animals in this study, we were not certain whether the observed difference was genuine. Therefore, we have compared the physical activity of WT that was unrelated to SPAK KI with the physical activity of SPAK KI and littermate WT. We have found that the SPAK KI had also spent longer in active physical state compared to unrelated WT mice and that there was no difference in physical activities of the two WT groups. This suggests that the observed difference in physical activity is genuine and that the inhibition of SPAK induces an increase in spontaneous locomotor activity.

Spontaneous locomotor activity is centrally regulated primarily by systems involved in energy balance, their regulation, and the control of food intake (Lenard and Berthoud 2008; Loprinzi et al. 2013). The important areas of the central nervous system (CNS) network that are involved are the corticolimbic circuits, the hypothalamus, and the caudal brainstem. It has been shown that electrical stimulation of the substantia nigra and the ventral tegmental area elevates spontaneous physical activity while lesions in the CNS can have a large impact on spontaneous physical activity in experimental animals (Rubia et al. 2014). On the other hand, abnormal CNS function in human subjects with attention deficit hyperactivity disorder has been linked to hyperactivity (Rubia et al. 2014). The WNK kinases behave like volume‐sensitive kinases that control SLC12 family members, and these SLC12 channels have been linked with pathological excitability and epilepsy. Bumetanide, a NKCC1 blocker, suppresses seizures and attenuates electrographic activity (Gautam et al. 2010). SPAK, ORS1, and WNK kinases have been also implicated in regulating the function of gamma aminobutyric acid (GABA) in the CNS (Watanabe and Fukuda 2015). It has been previously reported that SPAK knock out (KO) mice were characterized by diminished spontaneous physical activity (Geng et al. 2010). This is not in keeping with our findings as we have observed the opposite effect on physical activity. There are some significant differences between the two studies. Here, we have used telemetry to measure physical activity, with continuous 24 h‐long monitoring in SPAK KI mice (in comparison to SPAK KO mice). Long‐lasting monitoring of physical activity by telemetry is accepted as an appropriate methodology to monitor spontaneous locomotor activity (Huetteman and Bogie 2009; Fowler and Kenny 2012 **)**.

One possible criticism of our study would be the small number of animals that were used. However, our analysis of the physical activity of WT mice unrelated to SPAK KI demonstrated that physical activity levels are stable across the groups of mice with relatively narrow range of values. The fact that we had statistical significance with only 3 mice per experimental groups (4 mice in case of unrelated WT) suggest that observed differences were genuine and considerable. Taken all together, it is reasonable to conclude that an increase in spontaneous locomotor activity is indeed as a result of decreased SPAK activity.

At the moment, we cannot comment on the mechanism underlying altered physical activity. However, it is clear that SPAK inhibition increases locomotor activity which has an impact on the heart rate and blood pressure. Thus, the observed effect of SPAK inhibition on locomotor activity deserves to be further investigated.

## Conflict of Interest

The authors declared no potential conflicts of interest with respect to the research, authorship, and/or publication of this manuscript.
